# Volatile responses of dwarf birch to mimicked insect herbivory and experimental warming at two elevations in Greenlandic tundra

**DOI:** 10.1002/pei3.10100

**Published:** 2023-02-08

**Authors:** Jolanta Rieksta, Tao Li, Cleo L. Davie‐Martin, Laurids Christian Brogaard Aeppli, Toke Thomas Høye, Riikka Rinnan

**Affiliations:** ^1^ Terrestrial Ecology Section, Department of Biology University of Copenhagen Copenhagen Denmark; ^2^ Center for Permafrost (CENPERM) Department of Geosciences and Natural Resource Management University of Copenhagen Copenhagen K Denmark; ^3^ Sichuan Zoige Alpine Wetland Ecosystem National Observation and Research Station Key Laboratory for Bio‐resource and Eco‐environment of Ministry of Education College of Life Sciences Sichuan University Chengdu China; ^4^ Department of Bioscience and Arctic Research Centre Aarhus University Aarhus C Denmark

**Keywords:** Arctic, biotic stress, dwarf birch, insect herbivory, methyl jasmonate, stress severity, volatile organic compounds

## Abstract

Plants release a complex blend of volatile organic compounds (VOCs) in response to stressors. VOC emissions vary between contrasting environments and increase with insect herbivory and rising temperatures. However, the joint effects of herbivory and warming on plant VOC emissions are understudied, particularly in high latitudes, which are warming fast and facing increasing herbivore pressure. We assessed the individual and combined effects of chemically mimicked insect herbivory, warming, and elevation on dwarf birch (*Betula glandulosa*) VOC emissions in high‐latitude tundra ecosystems in Narsarsuaq, South Greenland. We hypothesized that VOC emissions and compositions would respond synergistically to warming and herbivory, with the magnitude differing between elevations. Warming increased emissions of green leaf volatiles (GLVs) and isoprene. Herbivory increased the homoterpene, (E)‐4,8‐dimethyl‐1,3,7‐nonatriene, emissions, and the response was stronger at high elevation. Warming and herbivory had synergistic effects on GLV emissions. Dwarf birch emitted VOCs at similar rates at both elevations, but the VOC blends differed between elevations. Several herbivory‐associated VOC groups did not respond to herbivory. Harsher abiotic conditions at high elevations might not limit VOC emissions from dwarf birch, and high‐elevation plants might be better at herbivory defense than assumed. The complexity of VOC responses to experimental warming, elevation, and herbivory are challenging our understanding and predictions of future VOC emissions from dwarf birch‐dominated ecosystems.

## Graphical abstract

Harsher abiotic conditions in tundra might not limit VOC emissions from dwarf birch, nor in response to the higher herbivore pressure predicted in future. The complexity of VOC responses are challenging our understanding and predictions of future VOC emissions from dwarf birch‐dominated ecosystems.

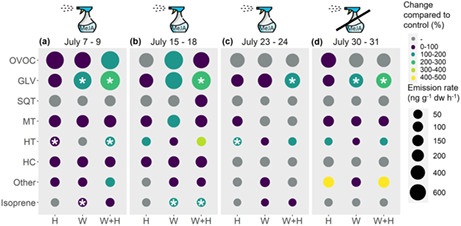

## 1 INTRODUCTION

Volatile organic compounds (VOCs) emitted from plants are specialized metabolites produced by plants. VOCs play important roles in plant physiology, signaling, and defense (Holopainen & Gershenzon, 2010), as well as in regulating plant primary metabolism (Erb & Kliebenstein, 2020). At the same time, VOCs are reactive trace gases which play an important role in atmospheric processes (Laothawornkitkul et al., 2009; Peñuelas & Staudt, 2010). More specifically, the oxidation of VOCs leads to the formation of secondary organic aerosol (SOA), which can scatter and absorb solar radiation, as well as participate in cloud formation (Zhao et al., 2017).

Climate change affects both the biotic and abiotic stressors of plants, such as temperature and insect herbivory, which can have significant impacts on plant VOC emissions (Faiola & Taipale, 2020; Peñuelas & Staudt, 2010). The effects may be more pronounced in the Arctic (IPCC, 2014; Rosenzweig et al., 2008; Walther et al., 2002), and studies have indeed already shown that warming directly and indirectly increases the emissions of plant VOCs from arctic vegetation (Faubert et al., 2010; Kramshøj et al., 2016; Lindwall et al., 2016). However, less is known about how biotic stress, such as insect herbivory pressure, which is expected to intensify due to the warming climate (Bale et al., 2002; Barrio et al., 2017; Kozlov et al., 2015), affects plant VOC emissions in the high latitudes.

Insect herbivory is a significant source of plant stress and vegetation damage that increases VOC emissions and alters VOC composition (Faiola & Taipale, 2020). In the Subarctic, increased insect herbivory pressure has been shown to enhance plant VOC emissions (Li et al., 2019; Rieksta et al., 2020, 2021; Ryde et al., 2021; Yli‐Pirilä et al., 2016). These herbivory‐induced VOC emissions contribute a substantial fraction of the total biogenic VOCs emitted into the atmosphere and therefore have the potential to affect chemical reactions in the atmosphere (Joutsensaari et al., 2015; Yli‐Pirilä et al., 2016; Zhao et al., 2017).

Two earlier studies indicate that plants exhibit stronger VOC responses to the combination of herbivory and warming than to either of these stressors alone (Li et al., 2019; Rieksta et al., 2021). These amplified VOC emissions with combined insect herbivory and warming may result from the following processes acting in tandem. First, both warming and herbivory can augment the expression of genes and the activity of enzymes involved in VOC biosynthesis (De Moraes et al., 2001; Dicke, 2009; Ghirardo et al., 2020; Kessler & Baldwin, 2001). Second, increased volatility and diffusion enhance the release of VOCs from storage compartments upon warming (Laothawornkitkul et al., 2009). In addition, insect feeding breaks the storage compartments within the leaves, which leads to enhanced release of VOCs from these compartments (Loughrin et al., 1994). However, a recent study by Rieksta et al. (2021) suggested that herbivory effects on plant VOC emissions are also modified by gradual changes in plant anatomical traits under climate change. Thus, acclimation of plants to the prevailing climate can challenge the understanding of how insect herbivory will affect plant VOCs in a changing climate. Furthermore, tundra ecosystems are highly heterogeneous and tundra plant responses to warming vary greatly (Bjorkman et al., 2018; Collins et al., 2021). Indeed, previous studies have indicated the unique specificity of tundra VOC emissions as a function of location and vegetation type (Angot et al., 2020; Ghimire et al., 2021; Kramshøj et al., 2016). Thus, field studies in other high‐latitude regions are needed to provide a more comprehensive understanding of the sources and drivers of VOC emissions from high‐latitude ecosystems.

Elevational gradients are great systems for assessing climate change effects because they act as ‘natural experiments’ with varying temperature, water availability, and lengths of the growing season (Körner, 2007). When coupled with experimental warming treatments that assess effects of shorter‐term temperature manipulations, elevational gradients allow for distinguishing these short‐term warming effects from the effects of long‐term warming via comparison of elevations (Sundqvist et al., 2020). Biotic interactions such as insect herbivory and associated plant defenses (e.g., VOC emissions) also respond to the abiotic changes along elevational gradients (Moreira et al., 2018). Harsher high‐elevation environments, with lower ambient temperatures, typically have higher wind exposure, water stress, and nutrient stress, and have been selected for lower insect herbivory, predation, diseases, and pollination than low‐elevation ecosystems (Moreira et al., 2018). Thus, stronger biotic interactions in low‐elevation plant communities have selected for higher induced and constitutive defenses, and the opposite is true for high‐elevation plants (Pellissier et al., 2016). However, contrasting or unclear relationships of induced and constitutive defenses between low‐ and high‐elevation communities have also been observed in other studies (Galmán et al., 2021; Rasmann et al., 2014).

VOC emissions are complex and consist of a wide variety of compounds (Dudareva et al., 2006). Different VOC compounds or groups have different ecological functions and exert different impacts on atmospheric chemistry (Dudareva et al., 2006; Laothawornkitkul et al., 2009; Peñuelas & Staudt, 2010). However, previous studies on elevational effects on plant VOC emissions have focused on a small fraction of VOCs, mainly terpenoids (Descombes et al., 2020; Moreira et al., 2018; Pellissier et al., 2016). Thus, more studies are needed to better understand the elevational effects on plant VOC emissions and their potential implications for both ecological and atmospheric processes.

We aimed to assess how climate warming and mimicked insect herbivory interact to affect plant VOC emissions in Greenlandic tundra. Mimicked herbivory was achieved using the plant hormone, methyl jasmonate (MeJA), which is known to modulate plant defenses against herbivory and has been successfully applied in other studies (Li et al., 2019; Papazian et al., 2019; Schuman et al., 2009). To assess how warming interacts with insect herbivory, we quantified dwarf birch (*Betula glandulosa*) VOC emissions with and without mimicked insect herbivory in a long‐term warming experiment placed at two elevations in Narsarsuaq, South Greenland. Overall, we expected lower VOC emission rates in the harsher environment of the high‐elevation site compared to the low‐elevation site. We also expected that mimicked insect herbivory would increase VOC emission rates and alter the VOC blends at both elevations due to the defensive functions of plant VOCs against insect herbivory. However, we hypothesized that responses to herbivory would be weaker at high elevation, due to the more relaxed plant defenses compared to low elevation. As climate warming can promote plant defenses via enhanced metabolism, we hypothesized that higher VOC emissions would result upon mimicked herbivory in the warming treatment, than in the ambient control treatment at both elevations.

## 2 MATERIALS AND METHODS

### 2.1 Study site and experimental design

The study was carried out in a long‐term warming experiment located in mesic tundra heath at two elevations in Narsarsuaq, South Greenland during the growing season of 2019. The low‐elevation site was located 50 m a.s.l. (61°11'N, 45°22'W), and the high‐elevation site was located 450 m a.s.l. (61°09'N, 45°23'W). The vegetation at both sites was dominated by grayleaf willow (*Salix glauca* L.) and dwarf birch (*Betula glandulosa* Michx.) (Lindén et al., 2022).

Each site hosts a warming experiment with eight blocks in which warming and ambient control plots are nested. The warming treatment was accomplished using transparent hexagonal open‐top chambers (OTCs) consisting of plexiglas material (1.14 mm thickness; eplastics.com) with an inside diameter of 1.4 m centered on an area of 2 × 2 m. The experiment started in the growing season of 2016, and since then, the OTCs have been set up every year at the beginning of the growing season (~May) and removed at the end of the growing season (~September).

### 2.2 Mimicked herbivory treatment

When herbivorous insects feed on plants, they often cause a varying degree of foliar damage. To avoid this variation, we used mimicked herbivory to allow for uniform treatments. Two healthy‐looking dwarf birch branches, growing approximately 50 cm apart within each plot, were selected (*n* = 32 plants/elevation). One branch was randomly subjected to mimicked herbivory treatment, while the other served as a control.

We used the volatile plant hormone, methyl jasmonate (MeJA), to mimic insect herbivory, following the method from Li et al. (2019). Previous studies, including one study in dwarf birth (*B. nana*), have consistently shown that exogenous application of MeJA induces VOC emissions similarly to feeding by chewing herbivores (Li et al., 2019; Waterman et al., 2019). The major difference is the green leaf volatiles (GLVs), which are synthesized by lipoxygenase (LOX) pathway upon mechanical leaf damage caused by, for example, leaf‐chewing insects, and is not responsive to MeJA application (Ameye et al., 2018; Scala et al., 2013).

Briefly, we enclosed a branch in a polyethylene terephthalate (PET) bag and applied 750 μL of 1 mM MeJA dissolved in water with a spray bottle; control branches were sprayed with water in the same manner. The PET bag was removed right after the MeJA spraying. MeJA treatment started on July 3 and July 5 at high and low elevations, respectively, and ended on July 22 and July 23, respectively (Figure S1). At each elevation, MeJA was applied on two consecutive days, followed by a one‐day break, prior to a third MeJA application. The same branches were sprayed with MeJA and measured throughout the VOC measurement weeks. The MeJA treatment was applied over three weeks, and in each week VOC measurements were conducted on the next day following the third MeJA application (Figure S1). The VOC measurement in the second week at the low‐elevation site was postponed by one day due to bad weather.

### 2.3 VOC measurements

**FIGURE 1:**
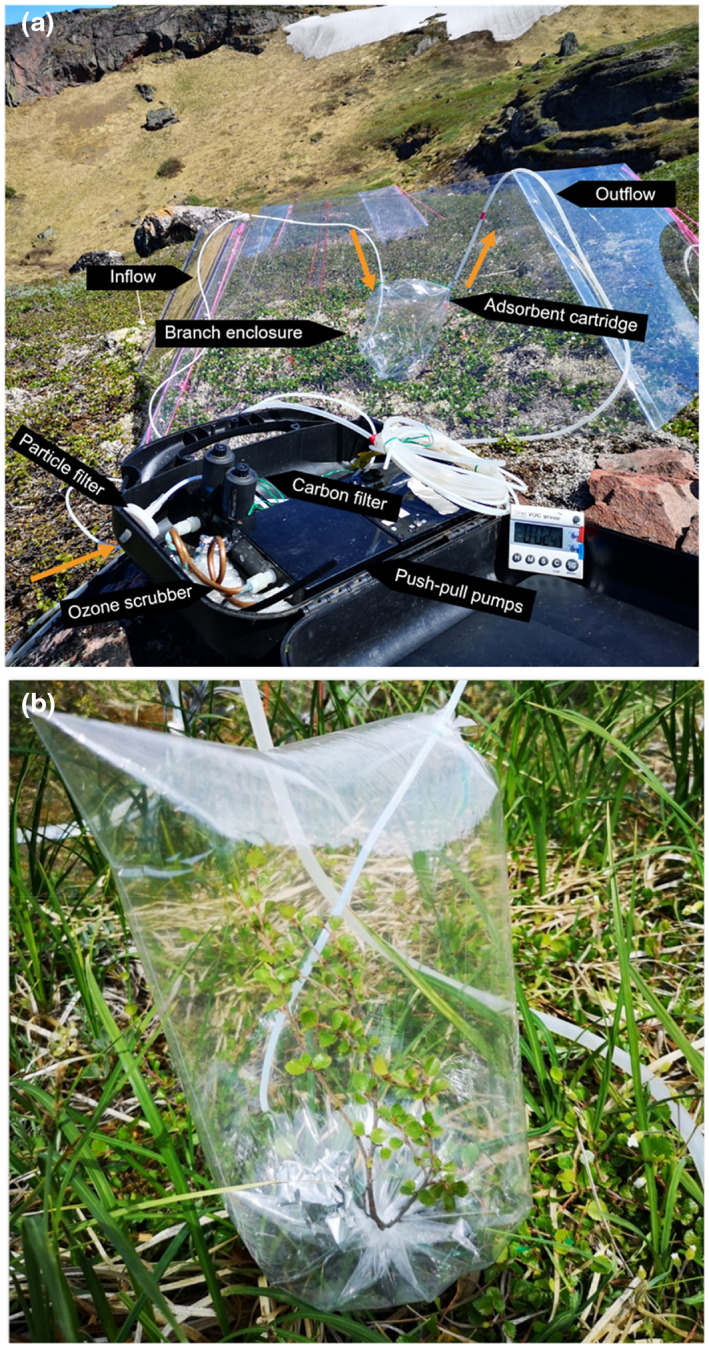
Overview of the VOC measurement technique in the field. (a) Custom‐built pull‐push system that is used for sampling VOCs. Black arrows indicate different parts of the system. Orange arrows indicate the air flow. (b) Polyethylene terephthalate (PET) bags are used as the branch enclosure.

We measured VOCs at high elevation on July 7, July 15, and July 23, and at low elevation on July 9, July 18, and July 24. To examine whether the potential herbivory effects remained after herbivory stopped, we also measured VOCs one week after the final MeJA application (July 30 at high elevation and July 31 at low elevation). VOCs were captured using the branch enclosure method described previously (Figure **1**; Vedel‐Petersen et al., 2015). Pre‐cleaned (120°C for 1 h) PET bags (25 × 38 cm) were used as enclosures through which clean air was circulated with pumps. VOCs were trapped from outgoing air in stainless steel adsorbent cartridges (150 mg Tenax TA, 200 mg Carbograph 1TD, Markes International Ltd, Llantrisant, UK). Each PET bag was ventilated for approximately 5 min before the measurement with an inflow rate of 1000 mL min^−1^. The incoming air was filtered for particles and background hydrocarbons, and scrubbed for ozone to avoid losses of the highly reactive VOCs (Kramshøj et al., 2016; Valolahti et al., 2015). Subsequently, the adsorbent cartridge was inserted into the corner of the PET bag and secured with wire. During the 20‐min sampling period, air was circulated through the enclosure with an inflow rate of 300 mL min^−1^ and an outflow rate of 200 mL min^−1^. The excess air leaked out of the opening where the branch entered the bag. After sampling, the cartridges were sealed with Teflon‐coated brass caps and stored at 5°C until analysis. To account for the compounds derived from sampling materials and the analytical system, one blank sampling was conducted in situ during each measurement day using the same approach but with empty PET bags. A new PET bag was used for each measurement.

After the last VOC measurement, we harvested all sampled branches, and the leaves were oven dried at 70°C for 72 h to determine dry mass, which was used for the calculation of emission rates.

### 2.4 VOC analysis

VOC samples were analyzed using gas chromatography–mass spectrometry (7890A Series GC coupled with a 5975C inert MSD/DS Performance Turbo EI System, Agilent Technologies, Santa Clara, CA) after thermal desorption at 250°C for 10 min (TD100‐xr, Markes International Ltd.). The carrier gas was helium (1.2 mL min^−1^), and the oven temperature was held at 40°C for 3 min, then raised to 210°C at a rate of 5°C min^−1^, and finally to 250°C at a rate of 20°C min^−1^, with a hold of 8 min. An HP‐5 capillary column (50 m length, 0.2 mm diameter, 0.33 μm film thickness, Agilent Technologies) was used for VOC separation.

Chromatograms were analyzed using PARADISe v. 3.8. software (Johnsen et al., 2017). Compounds were identified using pure standards, when available, or tentatively identified against a mass spectra in the 2014 NIST Mass Spectral Library. VOC concentrations were quantified using external standards. Quantification of VOCs for which pure standards were unavailable was achieved using the most structurally related standard compound (Methods S1).

Compounds that are known to arise from plastics, the analytical system, sorbent tubes, or personal care products were removed from the dataset, including siloxanes, phthalates, dibutyl adipate, 2‐ethylhexyl salicylate, and homosalate. Because the adsorbent cartridges cannot reliably trap lighter VOCs, we also removed compounds smaller than isoprene. We categorized compounds into the following groups: isoprene, monoterpenes (MTs), sesquiterpenes (SQTs), green leaf volatiles (GLVs), hydrocarbons (HCs), oxygenated VOCs (OVOCs), and other VOCs. VOC emission rates were calculated according to Ortega and Helmig (2008) and expressed on the basis of leaf dry mass (ng g^−1^ dw h^−1^).

### 2.5 Environmental variables

To examine air temperature, relative humidity, and photosynthetically active photon flux density (PPFD) during the VOC measurements, we placed iButtons (Hygrochron, Maxim Integrated) and Photosynthetic Light (PAR) Smart Sensors (S‐LIA‐M003, Onset Computer Corporation) coupled with a HOBO Micro Station Data Logger (H21‐002, Onset Computer Corporation) at the height of the dwarf birch in the warming and control plots (Table S1). Air temperature and relative humidity were also measured inside the branch enclosures during VOC sampling. In addition, we measured the average soil moisture (*n* = 5) around the base of each plant with a ML3 ThetaProbe soil moisture sensor (Delta‐T Devices Ltd.).

### 2.6 Statistical analysis

All statistical analyses were performed using the R statistical framework—version 4.0.3 (R Core Team, 2021). To assess the effects of herbivory, warming, and elevation on VOC emissions and their interactions, we used linear mixed‐effect models (LMM) fitted with maximum likelihood (ML) using the ‘lmer’ function from the *lme4* package (Bates et al., 2014). We performed LMMs with total VOCs, VOC groups, or individual VOC emission rates as the response variables; warming (ambient, treatment), herbivory (control, herbivory), elevation (low, high), measurement week (July 7–9, July 15–18, July 23–24, July 30–31), and their interactions were entered as fixed factors; block, plot, and plant were nested random factors in the LMMs. We used backward model reduction method using function ‘drop1’ and chi‐squared test for model selection and simplified the models by removing statistically non‐significant interactions (statistical significance level for keeping interactions in the main model was set at *p* < .25, Table S2).

To assess the main effects (herbivory, warming, elevation), we used the ‘ANOVA’ function from the *LmertTest* package (Kuznetsova et al., 2017). Parameter estimates between the ambient control and warming treatments, and between the herbivory treatments were obtained from estimated marginal means (EMMs) using function ‘emmeans’ from the *emmeans* package (Lenth et al., 2020). For post‐hoc pairwise comparisons, we used the ‘contrasts’ function for the differences between the main effects of warming herbivory, elevation, and week, and Dunnett's test for comparing the treatments (herbivory [H], warming [W], and warming + herbivory [W + H]) to the ambient control (A).

To assess how herbivory, warming, and elevation altered the VOC blend composition and to identify compounds important for separating the blends, we ran the supervised machine learning algorithm, Random Forests (RF) (Breiman, 2003), with treatment (A, H, W, W + H) and elevation as the response variables and relative proportions of the individual VOCs as the predictor variables (Breiman, 2003). RF was run using the ‘randomForest’ function from the *randomForest* package. RF reveals if samples fall into distinct clusters and if so, how the clusters are related to a certain treatment (warming, herbivory, and elevation). If RF revealed clear clustering using treatment + elevation as the response variables, we performed separate RF analysis, where herbivory, warming, or elevation was entered as the response variable, in order to obtain a list of the most important individual VOCs contributing to the differences between the VOC blends from the different treatments and to obtain out‐of‐bag (OOB) error rates. More specifically, the RF was run with all data together, each elevation separately, each measurement week separately, and each measurement date separately. We drew *N*_tree_ = 100,000 bootstrap samples with *m*_try_ = 12 variables (VOCs) randomly selected at each node. Supervised RF returned OOB error rates and confusion matrices for each classification (Breiman, 2001, 2003). Furthermore, we obtained the importance of contribution of each VOC to the VOC blend composition differences, which was expressed as the mean decrease in accuracy (MDA). Higher MDA values mean that a specific VOC has higher importance in separating the VOC blends. We used Welch's t‐test to evaluate differences between the emission rates of VOCs with the highest MDA for the herbivory treatment and elevation.

## 3 RESULTS

### 3.1 Environmental conditions during the experiment

The abiotic factors during the VOC measurement period differed between the two elevations. Ambient temperature was, on average, 2.8 ± 0.6°C (mean ± SE) lower at high elevation compared to low elevation (*p* < .001; Table S1). PPFD was 18% higher at high elevation (*p* = .01; Table S1), but this difference was not consistent between measurement weeks. Soil moisture was 140% higher at low than high elevation (*p* < .001; Table S1).

During VOC measurements, the OTCs increased the canopy‐level air temperature by an average of 6.2 ± 0.6°C at high elevation (*p* < .001) and 4.2 ± 0.6°C at low elevation (*p* < .001; Table S1). The OTCs tended to decrease the PPFD by 12% at high elevation (*p* = .07; Table S1) and did not change the PPFD at low elevation (*p* = .85). At low elevation, soil moisture was 15% lower in the warming treatment than in the ambient controls (*p =* .07), but did not change in response to warming at high elevation (*p* = .9; Table S1).

### 3.2 Elevation effects on VOC emissions

**FIGURE 2:**
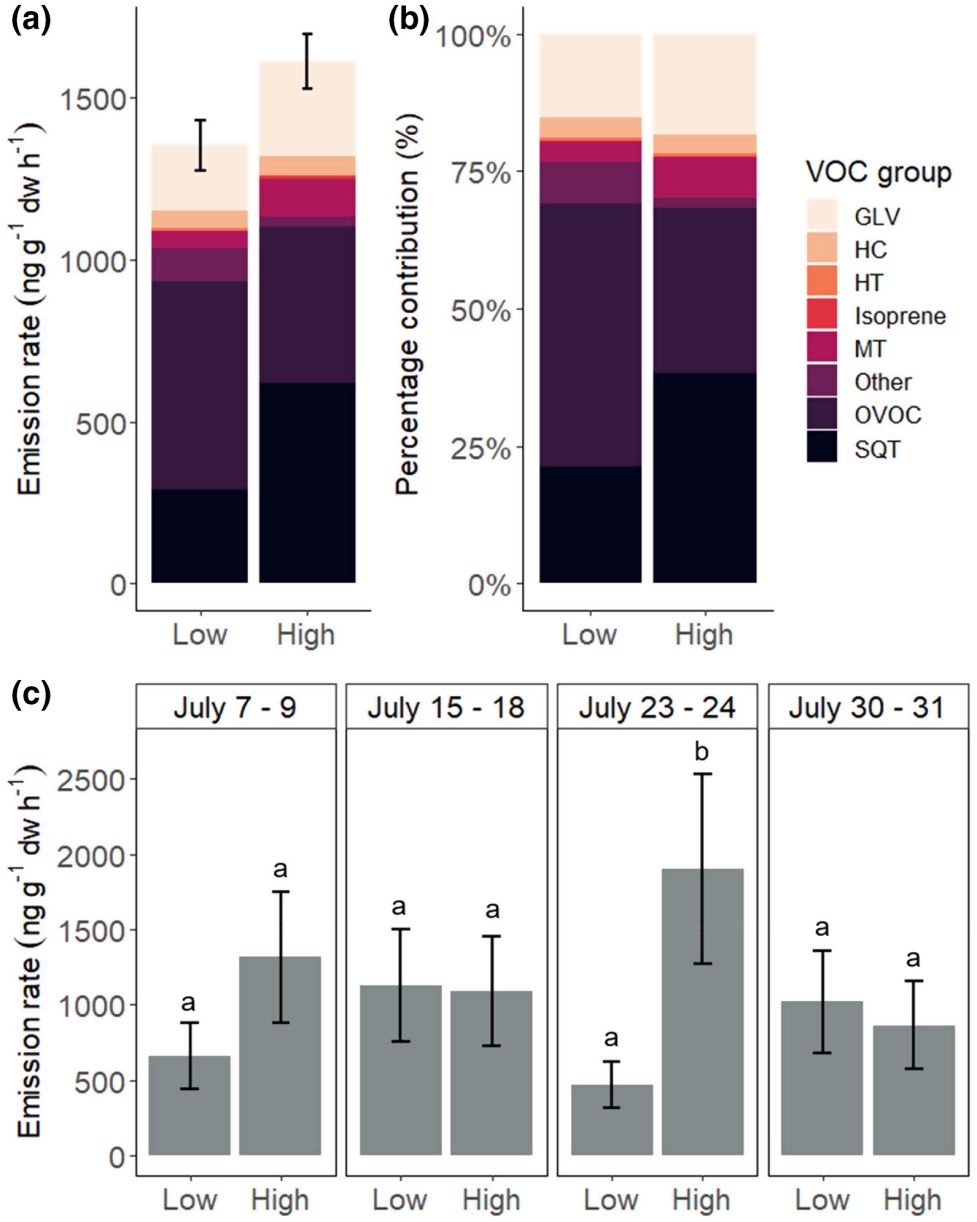
Emission rates and VOC blend composition for dwarf birch under ambient conditions at high and low elevations. (a) Emission rates (ng g^−**1**^ dw h^−**1**^) for each VOC group are stacked, and the standard errors of the mean are shown for total VOC emissions. The higher emission rates at high elevation are driven by the measurement campaign on July 23–24, when low elevation had lower ambient temperature (14°C) compared to high elevation (17°C) (see c). (b) Percentage contributions of each VOC group to the total VOC emissions. (c) Back‐transformed estimated marginal means (EMMs) of emission rates for total VOCs at two elevations for each measurement week. Letters indicate results from Tukey's test (*p* < .05). VOC groups: GLV (green leaf volatiles), HC (hydrocarbons), HT (homoterpene, (E)‐DMNT), MT (monoterpenes), Other (other VOCs), OVOC (oxygenated VOCs), and SQT (sesquiterpenes).

VOC emissions from dwarf birch in the ambient control were dominated by OVOCs, GLVs, and sesquiterpenes (Figure 2a,b). OVOCs comprised 48% of the total VOC emissions at low elevation, and 30% at high elevation, while GLVs contributed 15% and 18% at low and high elevation, respectively (Figure **2** **b**). Sesquiterpenes had lower contributions to the total VOC blend at low elevation (21%) than at high elevation (38%). Under 10% of the total VOCs were monoterpenes. The homoterpene, (E)‐4,8‐dimethyl‐1,3,7‐nonatriene ((E)‐DMNT), isoprene, hydrocarbons, and other VOCs were minor contributors to the emission profile of dwarf birch (<4%) (Figure 2b).

The emission rates of total VOCs and most VOC groups, that is, GLVs, monoterpenes, and sesquiterpenes, did not differ between the elevations during all measurement weeks (Figure 2c). The only exception was July 23–24, when the low‐elevation site had a lower ambient temperature (14°C) and thus a lower VOC emission rate than the high‐elevation site (17°C) (*p* < .05, elevation × week interaction; Table S3). (E)‐DMNT was the only VOC that had consistently higher emission rates at high elevation compared to low elevation, both when averaged across weeks and within each measurement week (Table S3).

**FIGURE 3:**
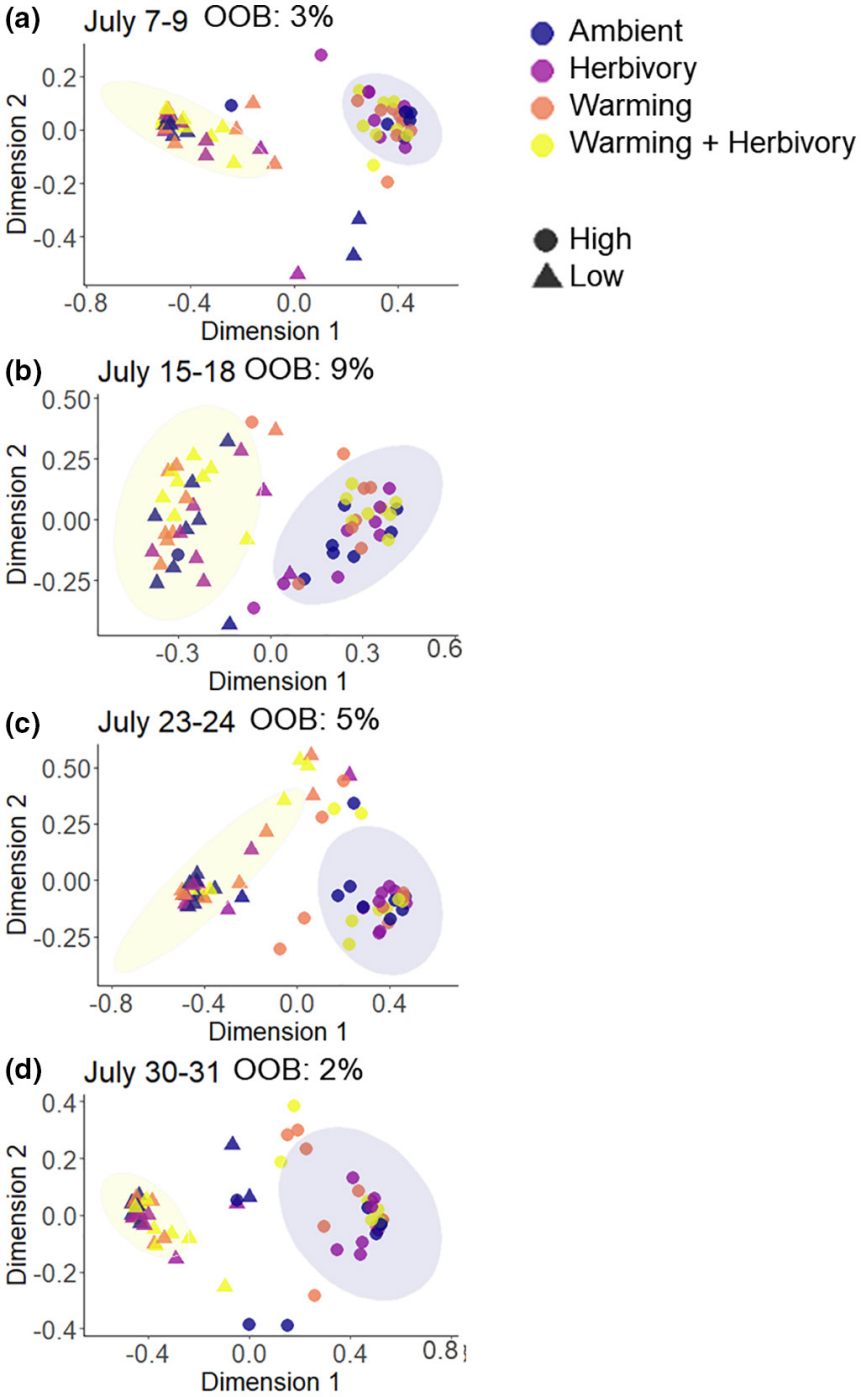
Supervised Random Forest (RF) analysis of the VOC blends emitted by dwarf birch in ambient control, mimicked herbivory, warming, and warming + mimicked herbivory treatments during each of the four measurement weeks at high and low elevation on (a) July 7–9, (b) July 15–18, (c) July 23–24, and (d) July 30–31. The out‐of‐bag (OOB) classification error is shown. The low error rates (OOB) show clear VOC blend differences between the elevations, but treatments could not be separated. The ellipses indicate the elevations ‐ high (blue color) and low (yellow).

The random forest analysis showed that the VOC blends were clearly separated by elevation across all measurements and during each measurement week (Figure 3). The VOCs that contributed most to the between‐elevation differences were methyl salicylate, α‐bourbonene, and (E)‐DMNT, all of which had higher emission rates at high than low elevation (*p* < .001).

### 3.3 Warming effects on VOC emissions

**FIGURE 4:**
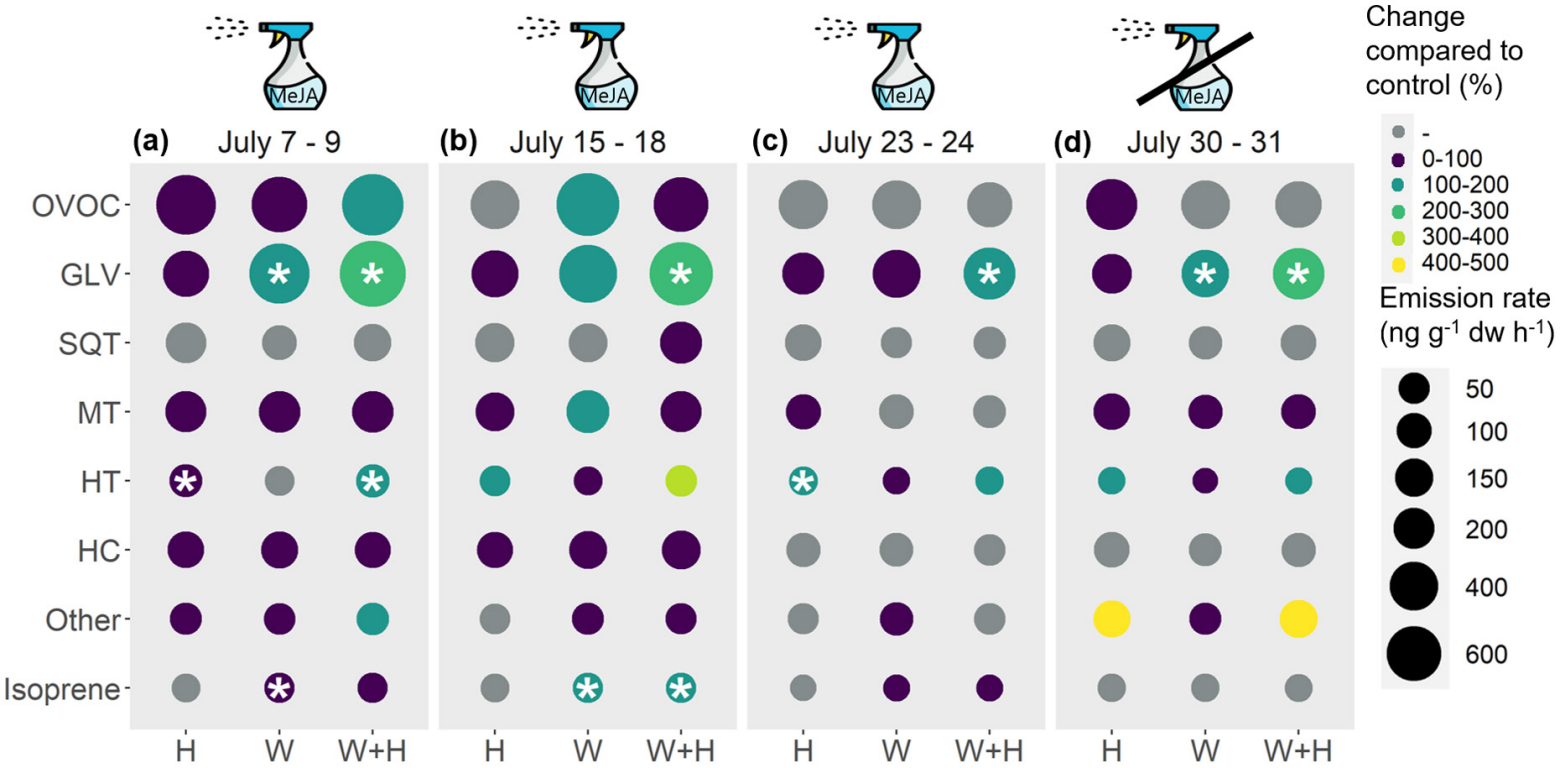
Heatmap showing VOC group responses to mimicked herbivory (H), warming (W), and their combination (W + H) in comparison to the ambient control for each measurement week (a) July 7–9; (b) July 15–18, (c) July 23–24, and (d) July 30–31 across both elevations. The first three periods correspond to measurements during mimicked insect herbivory treatment (MeJA application), while the last period corresponds to the measurement one week after mimicked insect herbivory treatment was stopped. The size of the circle represents the emission rate (ng g^−**1**^ dw h^−**1**^), and the color represents the percentage increase in the emission rate compared to the ambient control. *p*‐values < .05 from Dunnett's test are shown with asterisks. VOC groups: GLV (green leaf volatiles), HC (hydrocarbons), HT (homoterpene, (E)‐DMNT), MT (monoterpenes), Other (Other VOCs), OVOC (Oxygenated VOCs), SQT (sesquiterpenes). The emission rates are presented as back‐transformed estimated marginal means presented across both elevations, i.e., adjusted for elevation. The predicted emission rates for each measurement date at each elevation and treatment can be seen in Table S4. VOC groups are ordered from highest to lowest emission rates.

**FIGURE 5:**
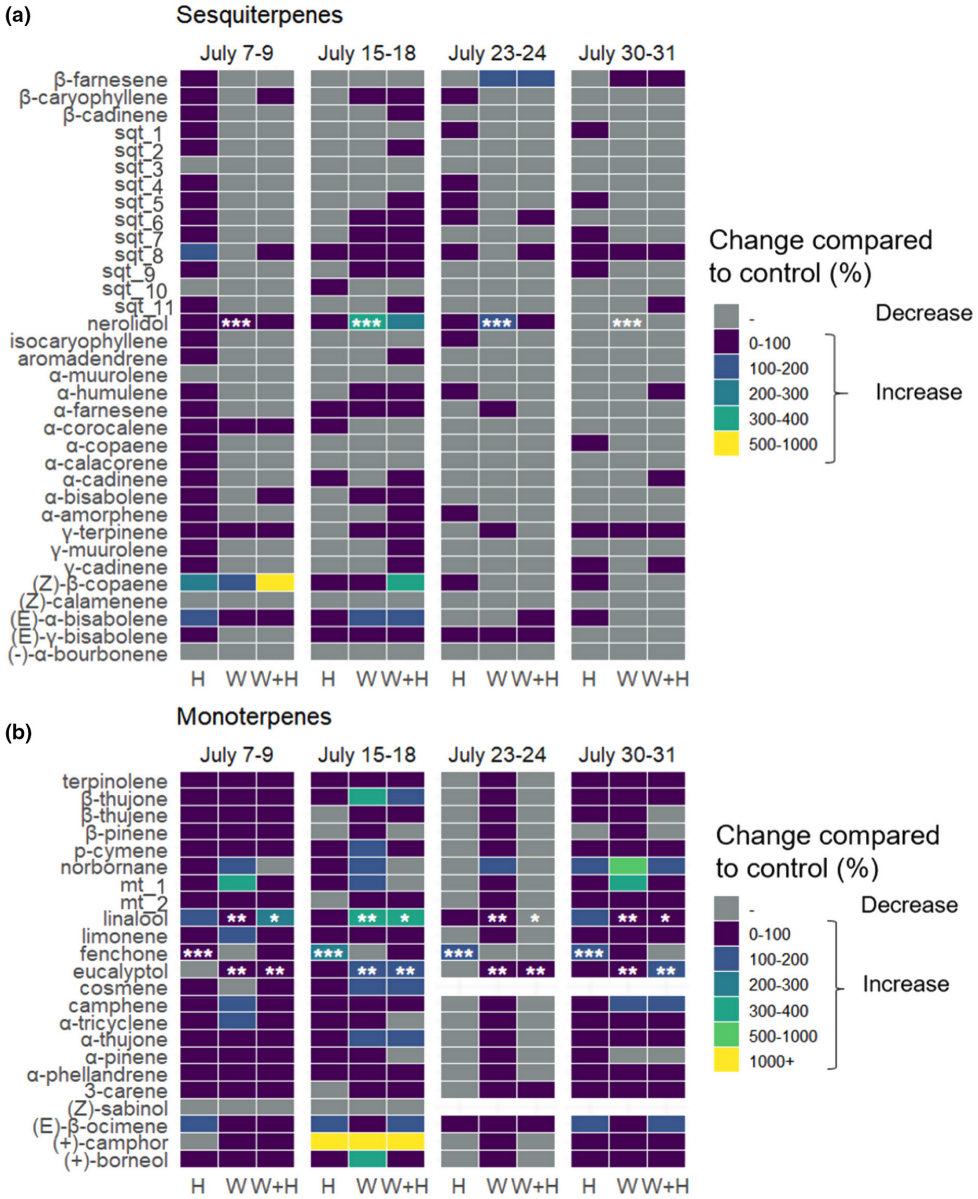
Heatmap for relative changes in emission rates of (a) individual sesquiterpenes and (b) monoterpenes in response to mimicked herbivory (H), warming (W), and warming+mimicked herbivory (W + H), compared to the ambient control across both elevations. Compounds are ordered from highest to lowest emission rates. The color shows percent change in the emission rate relative to the ambient control. Statistically significant differences (*p < .05, **p < .001, ***p < .0001, Dunnett's test) are denoted with asterisks. VOCs are presented in reversed alphabetical order.

We found strong warming effects on GLVs and isoprene at both elevations. Warming increased the GLV emissions by 180% across all measurements. More specifically, the GLV emissions increased from 121 ± 20 ng g^−**1**^ dw h^−**1**^ in ambient control to 340 ± 60 ng g^−**1**^ dw h^−**1**^ in warming treatment (*p* < .001). Isoprene emissions increased by 40% (ambient control: 3 ± 0.4 ng g^−**1**^ dw h^−**1**^, warming: 4 ± 6 ng g^−**1**^ dw h^−**1**^, *p* = .05; Figure 4). Monoterpene (ambient control: 48 ± 10 ng g^−**1**^ dw h^−**1**^, warming: 54 ± 12 ng g^−**1**^ dw h^−**1**^, *p* = .7) and homoterpene emissions (ambient control: 4 ± 0.8 ng g^−**1**^ dw h^−**1**^, warming: 4 ± 0.8 ng g^−**1**^ dw h^−**1**^, *p* = .9) did not change in response to warming (Figure 4a–d). Warming consistently, although not significantly, suppressed the emissions of total sesquiterpenes (Table S4) and individual sesquiterpenes (Figure 5a) at high elevation, but did not affect sesquiterpene emissions at low elevation. Furthermore, we found that the VOC blends could not be distinguished between the ambient control and warming treatments (Figure S2).

### 3.4 Effects of mimicked herbivory alone and in combination with warming and elevation

**FIGURE 6:**
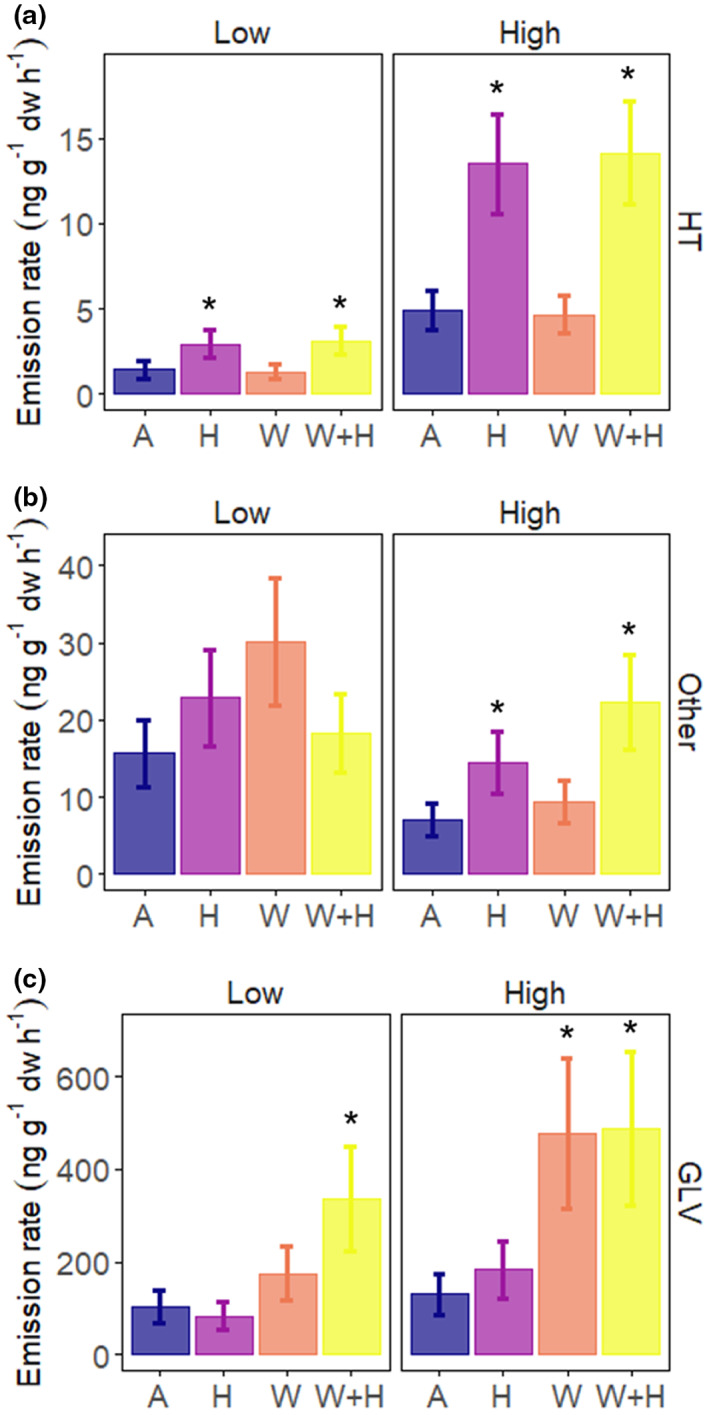
Estimated marginal means (EMMs) from linear mixed‐effects models showing the effects of warming (W), mimicked herbivory (H), and their combination (W + H) on VOC emissions at two elevations. (a) Homoterpene, (E)‐DMNT (HT), (b) Other VOCs (Other), (c) green leaf volatiles (GLV). The emissions are presented as back‐transformed EMMs and their associated standard error. Asterisks indicate differences from ambient control within each elevation (*p < .05, Dunnett's test).

Two VOC groups responded to mimicked herbivory: the homoterpene, (E)‐DMNT (*p* < .001; Figure 6a), and other VOCs, which comprised of three compounds with benzene rings, such as indole, and three unidentified VOCs (*p* = .05; Figure 6b). (E)‐DMNT was the only compound whose emission rates increased following mimicked herbivory treatment at both elevations, even one week after herbivory treatment (*p* = .06, herbivory × week interaction). On average, the emissions of (E)‐DMNT increased from 3 ± 0.5 ng g^−**1**^ dw h^−**1**^ in control treatment to 7 ± 1 ng g^−**1**^ dw h^−**1**^ in herbivory treatment. However, the response was stronger at high elevation (190% increase) than at low elevation (120% increase) (*p* = .08, herbivory × elevation interaction; Figure 6a and Figure 4a,b). Furthermore, (E)‐DMNT responded to herbivory to the same extent in the ambient and warming treatments at both elevations (Figure 6a).

Unlike (E)‐DMNT, the other VOCs only responded to mimicked herbivory at high elevation (F_1,229_ = 5.1, *p* = .02, herbivory × elevation interaction) and only one week after the mimicked herbivory treatment (F_3,229_ = 5.5, *p* = .001, herbivory × week interaction), when herbivory increased the emissions by 360% (t_187_ = 4.2, *p* < .0001). We also found a 220% increase in GLV emissions in response to the combination of herbivory and warming at low elevation (F_1,148_ = 1.7, *p* = .20; warming × herbivory × elevation interaction; Dunnett's test, *p* = .05; Figure 6c). In contrast, the monoterpenes (F_1,29_ = 0.2, *p* = .7) and sesquiterpenes (F_1,29_ = 0.4, *p* = .51) did not respond to mimicked herbivory (Figure 2a,b).

Individual VOCs responded inconsistently to herbivory and warming, with positive, negative, or no response was observed (Figure 5a,b; Figures S3 and S4). For example, while limonene, linalool, and (E)‐β‐ocimene emissions did not increase in response to herbivory (Figure 5b), the emission rate of fenchone increased by 60–240% depending on the measurement week (Dunnett's test, *p* < .001; Figure 5b).

Overall, the random forests analysis revealed no differences in the VOC blend compositions in response to herbivory (Figure S2). When assessing the measurement weeks separately, we only found clear herbivory effects on the VOC blend on the first measurement week at low elevation (Figure S5). Two esters, an unidentified ester and atraric acid, contributed most to the separation between control and herbivory VOC blends (Figure S5), as their emission rates were increased by mimicked herbivory (unidentified ester, t_16_ = −3.6, *p* = .003; atraric acid, t_21_ = −2.1, *p* = .04).

## 4 DISCUSSION

Our study, conducted at two elevations in the Greenlandic tundra, revealed that VOC emissions from the dwarf birch, *B. glandulosa*, were dominated by OVOCs, GLVs, and sesquiterpenes. The emission rates of most VOC groups were similar between harsher and colder high‐elevation site, and warmer and milder low‐elevation site during all measurement weeks. Homoterpene, (E)‐DMNT was the only VOC that had consistently greater emission rates at high than low elevation during all measurement weeks. Overall, VOC blends could not be separated by herbivory, warming, and combined treatments, but were clearly and consistently separated by elevation. Warming amplified GLV emissions and tended to suppress sesquiterpene emissions at high elevation. Mimicked insect herbivory using methyl jasmonate (MeJA) increased emissions of several individual VOCs, including (E)‐DMNT, indole, and few benzenoids. Responses upon herbivory were stronger at the colder high‐elevation site. Typical herbivory‐induced VOC groups—monoterpenes and sesquiterpenes, as well as individual VOCs—responded to herbivory and combined herbivory and warming treatments in an inconsistent way.

### 4.1 Mimicked herbivory effects and the combined effects of herbivory with warming and elevation

Our study showed that mimicked herbivory on the dwarf birch, *B. glandulosa*, strongly increased emissions of the homoterpene, (E)‐DMNT, indole, and a few unidentified compounds. (E)‐DMNT consistently increased in response to mimicked herbivory at both elevations, but in contrast to our expectation, the increase upon herbivory was larger at high elevation (190%) compared to low elevation (120%). Our results are in agreement with those of Rasmann et al. (2014), who found stronger induction of VOC emissions from bush vetch at high elevation upon exogenous application of jasmonic acid. Rasmann et al. (2014) suggested that, in some cases, the stronger induction could be because plants at high elevation might be better at modulating indirect defenses, such as predator recruitment.

Contrasting or absent herbivory effects on VOC emissions have been reported in other high‐latitude field studies (Ghimire et al., 2021; Li et al., 2019; Rieksta et al., 2021). Similarly, we did not observe clear herbivory effects on total sesquiterpene nor monoterpene emissions in this study. However, we did find that a few individual monoterpenes responded to mimicked herbivory both during, and one week after, the mimicked herbivory treatment. Several other individual VOCs responded inconsistently to mimicked herbivory, with positive, negative, or no responses observed.

The complex responses to mimicked herbivory might reflect the fact that in nature, plants often face multiple stresses and respond differently to single stresses compared to the combination of multiple stresses (Dicke, 2009; Holopainen & Gershenzon, 2010; Schweiger et al., 2014). Other factors not measured in this study could have also contributed to the absence of herbivory effects on VOC emissions. For example, high environmental spatial heterogeneity in tundra ecosystems (Bjorkman et al., 2018) resulting in genetic differentiation and phenotypic plasticity within species, or extensive hybridization of dwarf birch (de Groot et al., 1997; Thórsson et al., 2007), may have contributed to high variations in VOC emissions and thus confounded the VOC responses to herbivory. To disentangle herbivory effects from other confounding factors (e.g., genetic variation), field and laboratory studies controlling for confounding factors are needed.

We found that the VOC blend compositions in control and mimicked herbivory treatments were largely similar and altered only during the first week of the mimicked herbivory treatment at low elevation. The clear blend differences early in the herbivory treatment period may be related to the early attraction of natural enemies during herbivore attack (Joo et al., 2018; Moreira et al., 2018). The similar blend compositions during other measurement weeks between control and herbivory‐treated branches might be because the control plants also emitted several VOCs typically associated with insect herbivory (indole, methyl salicylate, (E)‐β‐ocimene, eucalyptol) (Pare & Tumlinson, 1997), which may be an indication of other stresses or herbivores present in the landscape.

GLVs are typical herbivory‐induced VOCs emitted upon cell membrane damage (Ameye et al., 2018; Scala et al., 2013). As our MeJA‐mimicked herbivory treatment was accomplished without physical leaf damage, which leaf‐chewing herbivores would cause, it did not alter the GLV emissions of dwarf birch. GLVs have also been shown to increase in response to abiotic stresses, such as cold, heat, or high irradiance (Copolovici et al., 2011; Loreto et al., 2006), or upon phytopathogen infection (Piesik et al., 2011; Ponzio et al., 2013). Species can emit GLVs constitutively in large quantities (Arey et al., 1991; König et al., 1995), and indeed, GLVs are one of the largest VOC groups emitted by dwarf birch (Rieksta et al., 2021; Rinnan et al., 2011). In our study, GLV emission rates increased by 70% in response to warming and by 220% in the combined treatment of warming and mimicked herbivory, indicating synergistic effects of warming and mimicked herbivory for GLVs at low elevation. This is in agreement with the earlier reported consistently increased GLV emissions under warming in tundra ecosystems (Li et al., 2019; Rieksta et al., 2021; Rinnan et al., 2011). The amplified emissions under combined herbivory and warming treatment in this study suggest that in rapidly warming high‐latitude birch forests, where insect herbivore pressure is predicted to increase, GLV emissions will contribute to a substantial fraction of the total VOCs emitted into the atmosphere. From the atmospheric perspective, many herbivory‐induced VOCs, including terpenoids, have high secondary organic aerosol (SOA) formation potentials (Faiola & Taipale, 2020; Taipale et al., 2021), while GLVs might suppress SOA formation (Mentel et al., 2013). Thus, enhanced GLV emissions in a warmer future Arctic with increased herbivory might dampen SOA formation.

### 4.2 Differences between elevations

Plant VOC emissions along elevation gradients are influenced by the shift in climatic conditions (Simin et al., 2022). Harsher high‐altitude environments with low ambient temperatures, high wind exposure, and water and nutrient stress can physiologically constrain VOC production (Blande & Glinwood, 2016). In our study, the high‐elevation site had lower soil moisture and ambient temperatures than our low‐elevation site.

We found clear VOC blend differences between the two elevations throughout the measurement weeks, with methyl salicylate, α‐bourbonene, and (E)‐DMNT being the most important individual VOCs separating the blends. Both methyl salicylate and (E)‐DMNT are important herbivory‐induced VOCs (Dicke et al., 1999), potentially suggesting that the differing VOC blends resulted from differences in biotic stressors, rather than phenological differences between elevations. Also, abiotic and biotic factors have shaped plant communities at different elevations over time and potentially modulated plant defense responses that resulted in distinguishable VOC blends between elevations.

VOC emissions are temperature‐dependent (Niinemets et al., 2004). Thus, lower temperatures at high elevation should lead to lower VOC emissions than at low elevation. However, in contrast to the VOC blends, the VOC emission rates did not differ substantially between elevations, except during July 23–24, when the higher temperatures at high elevation resulted in overall higher VOC emissions. The only exception was the homoterpene, (E)‐DMNT, as it was consistently emitted at higher rates at high than low elevation within each measurement week. Similar emission rates between elevations are in contrast with the results of Rasmann et al. (2014), who found lower constitutive VOC emissions from bush vetch at high elevation than at low elevation. Furthermore, our result of similar emission rates of GLVs between the elevations is also in contrast with a recent study by Simin et al. (2022), who measured VOC emissions of dwarf birch over the growing season and found 235% higher GLV emissions at high elevation in the same study site and growing season of 2019. The contrasting VOC emission responses between studies in the same location might be due to high variation of tundra VOC emissions during the growing season (Baggesen et al., 2021). However, other environmental factors, such as light intensity and soil moisture, might offset the elevation‐associated temperature effects on VOC emissions. The low‐elevation site had 140% higher soil moisture than the high‐elevation site. Consequently, reduced transpiration at high elevation, due to drier soil conditions, could have contributed to the decoupling of the leaf temperature from the ambient air temperature and resulted in higher leaf temperatures and thus higher VOC emissions (Ambebe & Dang, 2009; Mao et al., 2004; Wang et al., 1998).

### 4.3 Conclusion

Our study in the Greenlandic tundra showed that while dwarf birch VOC blends differed between elevations, the contrasting environments with complex differences between abiotic and biotic drivers of emissions had largely similar VOC emission rates. This finding suggests that plants in harsh high‐elevation environments might be a higher source of VOC emissions than previously assumed. Mimicked herbivory increased emissions of the homoterpene, (E)‐DMNT, and some unidentified VOCs in the high‐elevation environment to a greater extent than at low elevation. However, several VOC groups that are often amplified in response to herbivory, did not respond to herbivory and warming at either elevation. Our results reveal the complexity and specificity of VOC responses to experimental warming, elevation, and herbivory, which challenges our ability to predict future VOC emissions from rapidly changing high‐latitude ecosystems. Taken together, these results suggest that harsher abiotic conditions at high elevations might not be a limitation to some tundra plants, like dwarf birch, in protecting them from the stronger herbivore pressure predicted for the future. In fact, our findings show that irrespective of warming, dwarf birch at high elevations might better modulate volatile defenses in response to insect herbivory than their low‐elevation neighbours.

## CONFLICT OF INTEREST STATEMENT

Authors declare no conflict of interest.

## Supporting information


Table S1.
Table S2.
Table S3.
Table S4.

Appendix S1.

## Data Availability

Supporting information and data supporting these findings can be found in Figshare https://figshare.com/s/95e503be3ef1f0b52fd8.
