# Supplementary material for: Volatile responses of dwarf birch to mimicked insect herbivory and experimental warming at two elevations in Greenlandic tundra
Source: Plant Environ Interact. 2023 Feb 8;4(1):23–35. doi: 10.1002/pei3.10100 (PMC10168049; doi:10.1002/pei3.10100)
Supplement: Supplementary file 2 — Appendix S1. [file PEI3-4-23-s002.docx]

## Supporting Information

Article title: Volatile responses of dwarf birch to mimicked insect herbivory and experimental warming at two elevations in Greenlandic tundra

Authors: Jolanta Rieksta, Tao Li, Cleo L. Davie-Martin, Laurids Christian Brogaard Aeppli, Toke Thomas Høye, Riikka Rinnan

The following Supporting Information is available for this article:

[**Table S1** Environmental variables recorded at canopy height in the ambient control (A) and warming treatment (W) during the VOC measurements at low and high elevations. PPFD (photosynthetically active photon flux density).](https://alumni-my.sharepoint.com/personal/lcm767_ku_dk/Documents/Desktop/Projects/Narsarsuaq/Word/2022/7_03062022/Submission/PEI/Revision/2022-12-16-JRieksta_Main%20text.docx#_Toc100652269)

[**Table S2** Model selection procedure for statistical analysis.](https://alumni-my.sharepoint.com/personal/lcm767_ku_dk/Documents/Desktop/Projects/Narsarsuaq/Word/2022/7_03062022/Submission/PEI/Revision/2022-12-16-JRieksta_Main%20text.docx#_Toc100652269) **Located in the "pei310100-sup-0001-Tables S1-S4.xlsx"**

**Table S3** The contrast estimates from estimated marginal means (EMMs) testing for differences in the emission rates between elevations for each VOC measurement week. VOC groups - GLV (green leaf volatiles), HC (hydrocarbons), HT (homoterpene (E)-DMNT), ISO (isoprene); MT (monoterpenes); SQT (sesquiterpenes), OVOC (oxygenated VOCs); Other (other VOCs), Total (total VOCs). The ratio contrast estimate between high and low elevation, standard error (SE), degrees of freedom (*df*), t-ratio, and *P-*value are shown for each measurement week (July 7-9, July 15-18, July 24-24, and July 30-31). **Located in the "pei310100-sup-0001-Tables S1-S4.xlsx"**

**Table S4** Back transformed estimated marginal means (EMM) from linear mixed effects models for dwarf birch *B. glandulosa-nana* complex VOC emission rates (ng g^-2^ dw h^-1^) for ambient control and warming treatment without herbivory (C) and with herbivory (H) at high and low elevation for each measurement week (July 7-9, July 15-18, July 24-24, and July 30-31). EMM, standard error (SE), degrees of freedom (*df*), lower and upper confidence intervals (CI), and *p*-value from Dunnett´s test are shown. VOC groups - GLV (green leaf volatiles), HC (hydrocarbons), HT (homoterpene (E)-DMNT), isoprene; MT (monoterpenes); SQT (sesquiterpenes), OVOC (oxygenated VOCs); Other (other VOCs), Total (total VOCs). **Located in the "pei310100-sup-0001-Tables S1-S4.xlsx"**

**Figure S1** Overview of the timing of methyl jasmonate (MeJA) application and subsequent VOC measurements at high and low elevation during July in the growing season of 2019.

**Figure S2** Supervised Random Forests (RF) analysis of the VOC blends emitted by dwarf birch in ambient control, mimicked herbivory, warming, and the warming + mimicked herbivory treatments during each of the measurement dates at two elevations.

**Figure S3** Heatmap for relative changes in emissions of **(a)** green leaf volatiles (GLVs) and **(b)** oxygenated VOCs (OVOC) to mimicked herbivory (H), warming (W), and their combination (W+H) during each measurement week and averaged across elevations.

**Figure S4** Heatmap for relative changes in emissions of **(a)** Other VOCs and **(b)** Hydrocarbons in response to mimicked herbivory (H), warming (W), and their combination (W+H) during each measurement week and averaged across elevations.

**Figure S5** Random Forests (RF) analysis of the VOC blends emitted by control and mimicked herbivory treated branches in ambient plots at low elevation on July 9. **(a)** Multidimensional scaling plot for RF on the VOC blends for control and herbivory treatment. **(b)** Top 10 VOCs with the highest mean decrease accuracy (MDA) from RF analysis of the VOC blends. The two VOCs that contributed the most to the separation between the control and herbivory VOC blends separation are denoted with asterisk (unidentified ester, t_16_= -3.6, *P* = 0.003; atraric acid, t_21_= -2.1, *P* = 0.04).

[**Table S1** Environmental variables recorded at canopy height in the ambient control (A) and warming treatment (W) during the VOC measurements at low and high elevations. PPFD (photosynthetically active photon flux density).](https://alumni-my.sharepoint.com/personal/lcm767_ku_dk/Documents/Desktop/Projects/Narsarsuaq/Word/2022/7_03062022/Submission/PEI/Revision/2022-08-12-JRieksta_Main%20text.docx#_Toc100652269)

|  |  |  | | **PPFD**  **(μmol m^−2^ s^−1^)** | | **Temperature**  **(°C)** | | **Relative humidity**  **(%)** | | **Soil moisture**  **(%)** | |
| --- | --- | --- | --- | --- | --- | --- | --- | --- | --- | --- | --- |
| **Elevation** | **Week** | | **Treatment** | **mean** | **SE** | **mean** | **SE** | **mean** | **SE** | **mean** | **SE** |
| High | July 7 - 9 | | A | 992 | 54.7 | 19.0 | 0.5 | 28.6 | 0.7 | 20.6 | 1.0 |
| Low | July 7 - 9 | | A | 1155 | 103.0 | 20.4 | 1.2 | 32.2 | 1.2 | 53.2 | 3.6 |
| High | July 7 - 9 | | W | 747 | 23.9 | 18.4 | 0.4 | 32.8 | 0.7 | 22.7 | 1.6 |
| Low | July 7 - 9 | | W | 1302 | 113.2 | 23.1 | 0.8 | 29.5 | 1.0 | 44.8 | 3.1 |
| High | July 15 - 18 | | A | 1355 | 21.2 | 14.8 | 0.4 | 59.7 | 0.9 | 15.8 | 1.0 |
| Low | July 15 - 18 | | A | 1364 | 133.5 | 21.3 | 0.7 | 52.7 | 2.0 | 40.4 | 3.0 |
| High | July 15 - 18 | | W | 1271 | 18.8 | 21.0 | 0.4 | 46.9 | 1.1 | 19.1 | 1.2 |
| Low | July 15 - 18 | | W | 1296 | 86.6 | 24.7 | 0.7 | 47.4 | 1.3 | 35.9 | 2.5 |
| High | July 23 - 24 | | A | 1399 | 33.7 | 17.4 | 0.3 | 55.7 | 2.7 | 13.4 | 1.0 |
| Low | July 23 - 24 | | A | 507 | 16.4 | 14.2 | 0.1 | 66.4 | 0.2 | 38.0 | 3.4 |
| High | July 23 - 24 | | W | 1240 | 38.8 | 29.0 | 0.6 | 39.2 | 0.9 | 16.1 | 1.1 |
| Low | July 23 - 24 | | W | 472 | 13.7 | 17.1 | 0.1 | 59.6 | 0.3 | 34.9 | 2.1 |
| High | July 30 - 31 | | A | 1273 | 24.7 | 14.3 | 0.2 | 64.2 | 0.9 | 15.9 | 1.2 |
| Low | July 30 - 31 | | A | 1086 | 115.1 | 21.5 | 0.6 | 47.5 | 1.7 | 34.6 | 3.3 |
| High | July 30 - 31 | | W | 1172 | 25.7 | 21.5 | 0.4 | 48.8 | 1.0 | 16.0 | 1.0 |
| Low | July 30 - 31 | | W | 1020 | 84.7 | 28.1 | 0.9 | 37.2 | 1.8 | 30.8 | 2.5 |
| High | Average | | A | 1255 | 26.6 | 16.2 | 0.3 | 53.1 | 1.8 | 16.4 | 0.6 |
| Low | Average | | A | 1028 | 64.0 | 19.6 | 0.5 | 49.2 | 1.7 | 41.5 | 1.9 |
| High | Average | | W | 1108 | 29.9 | 22.0 | 0.5 | 42.2 | 0.9 | 18.5 | 0.7 |
| Low | Average | | W | 1023 | 58.8 | 23.3 | 0.6 | 44.3 | 1.5 | 36.6 | 1.4 |

**
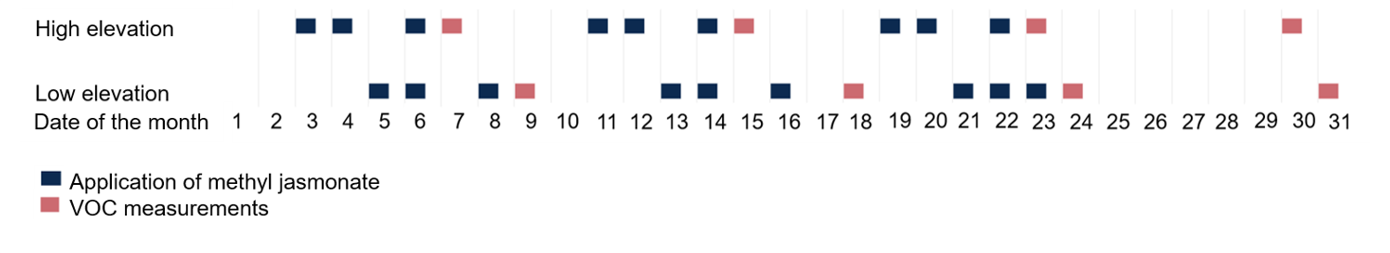
Figure S1** Overview of the timing of methyl jasmonate (MeJa) application and subsequent VOC measurements at high and low elevation during July in the growing season of 2019. Methyl jasmonate was applied for three days prior to VOC measurements.

**
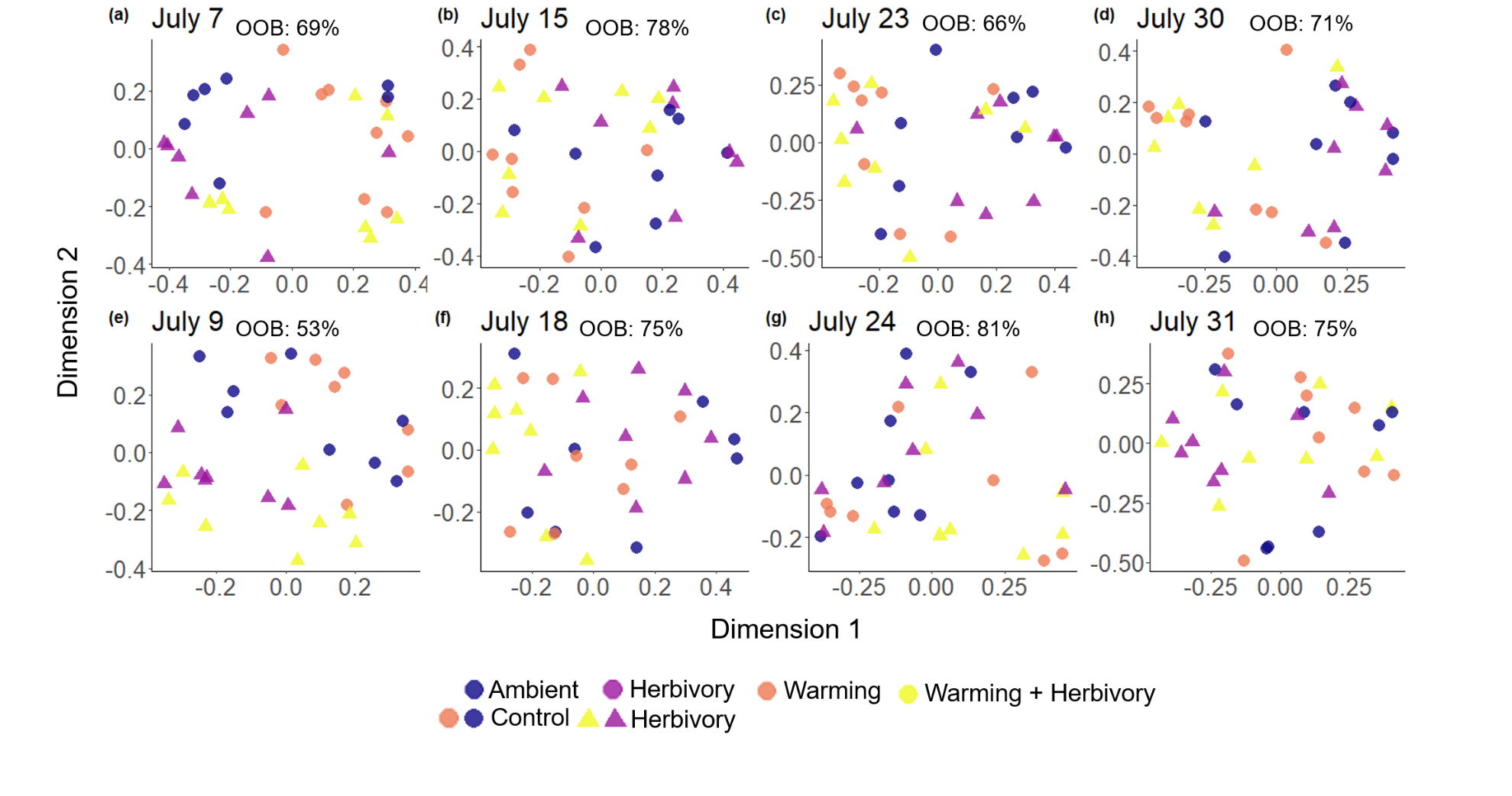
**

**Figure S2** Supervised Random Forests (RF) analysis of the VOC blends emitted by dwarf birch in ambient control, mimicked herbivory, warming, and the warming + mimicked herbivory treatments during each of the measurement dates at two elevations: **(a)** July 7, **(b)** July 9, **(c)** July 15, **(d)** July 18, **(e)** July 23, **(f)** July 24, **(g)** July 30, and **(h)** July 31. High elevation (July 7, July 15, July 23, July 30); low elevation (July 9, July 18, July 24, July 31). The out-of-bag (OOB) classification error is shown.

**
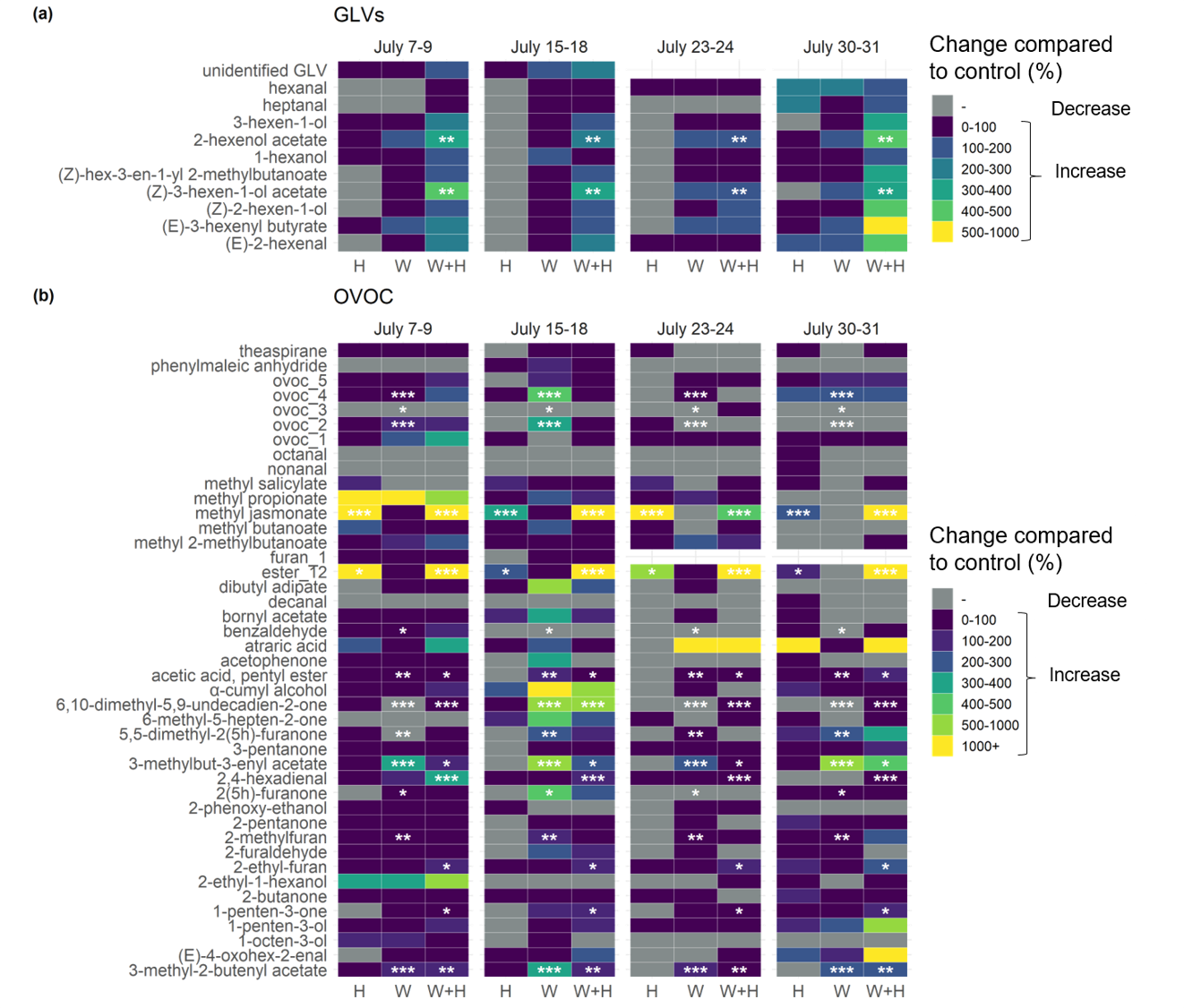
Figure S3** Heatmap for relative changes in emissions of **(a)** green leaf volatiles (GLVs) and **(b)** oxygenated VOCs (OVOC) to mimicked herbivory (H), warming (W), and their combination (W+H) during each measurement week and averaged across elevations. The colour shows percent change in the emission rate relative to the ambient control. Statistically significant differences (**P*<0.05. ***P*<0.001, ****P*<0.0001, Dunnett’s test) are denoted with asterisks.

**
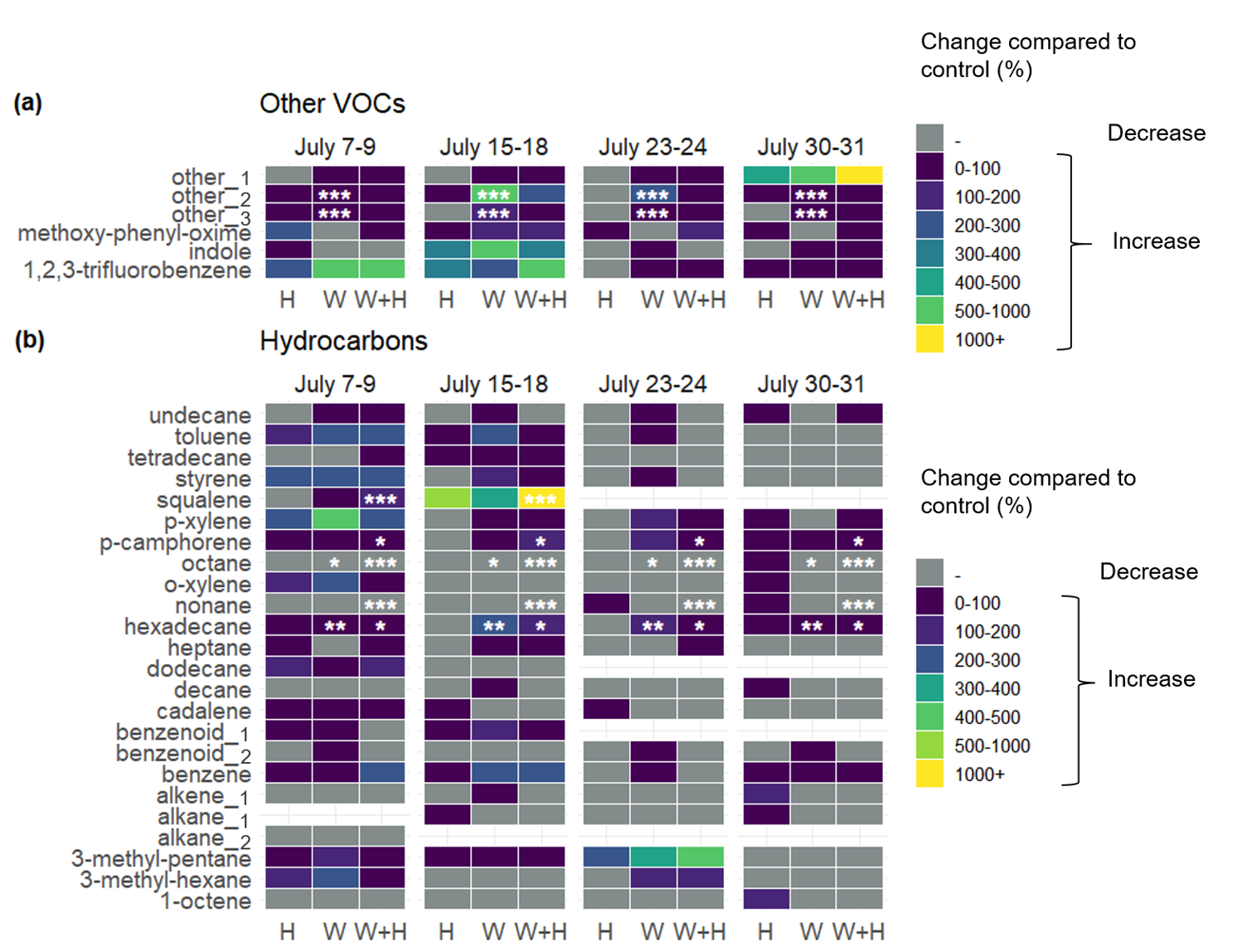
**

**Figure S4** Heatmap for relative changes in emissions of **(a)** Other VOCs and **(b)** Hydrocarbons in response to mimicked herbivory (H), warming (W), and their combination (W+H) during each measurement week and averaged across elevations. The colour shows percent change in the emission rate relative to the ambient control. Statistically significant differences (**P* < 0.05***P* < 0.001, ****P* < 0.0001, Dunnett’s test) are denoted with asterisks.
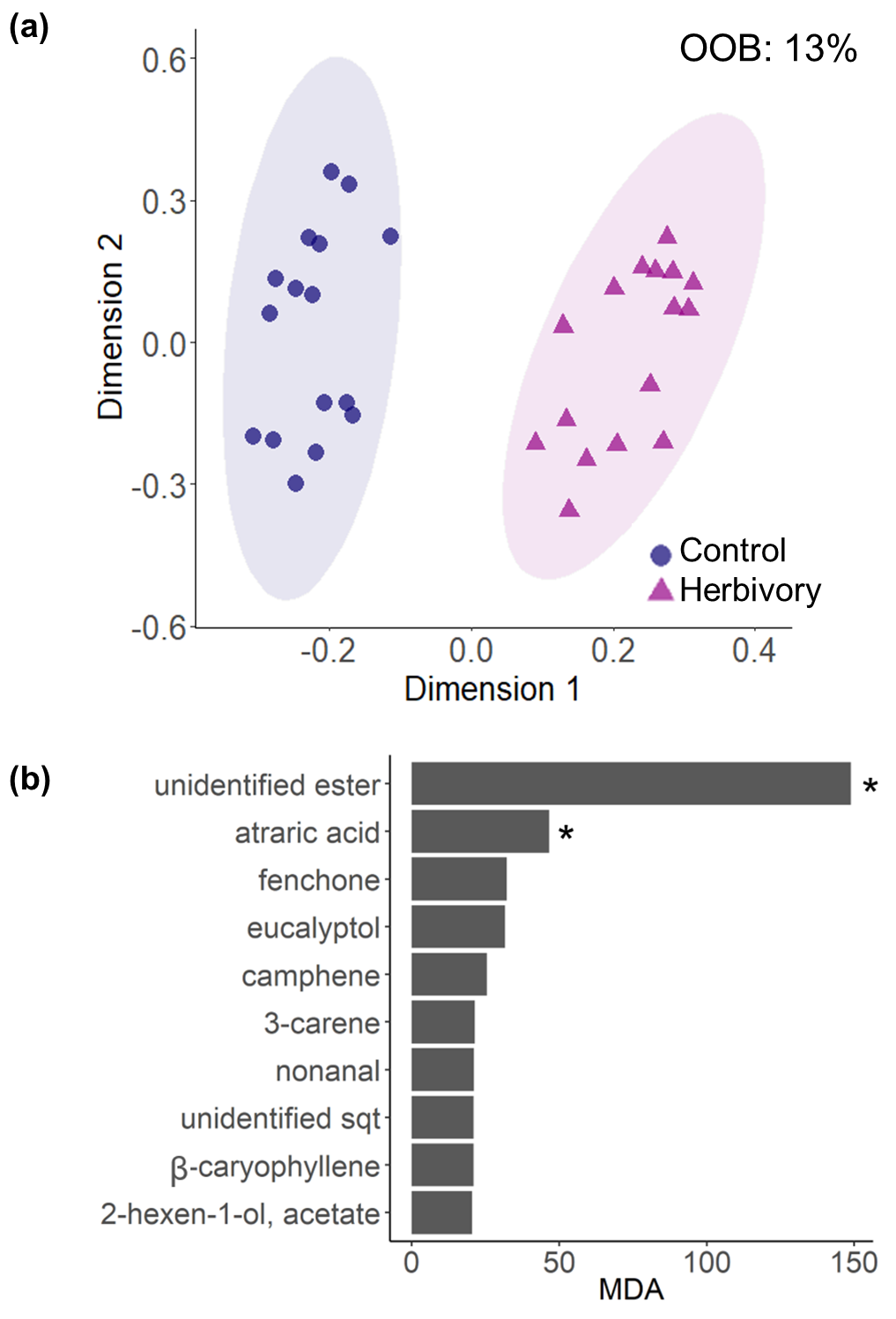


**Figure S5** Random Forests (RF) analysis of the VOC blends emitted by control and mimicked herbivory treated branches in ambient plots at low elevation on July 9. **(a)** Multidimensional scaling plot for RF on the VOC blends for control and herbivory treatment. **(b)** Top 10 VOCs with the highest mean decrease accuracy (MDA) from RF analysis of the VOC blends. The two VOCs that contributed the most to the separation between the control and herbivory VOC blends separation are denoted with asterisk (unidentified ester, t_16_= -3.6, *P* = 0.003; atraric acid, t_21_= -2.1, *P* = 0.04).

**Methods S1** PARADISe software VOC classification. VOC group, its abbreviations, VOC classes that are included in the VOC group, as well as specifics and associated standards are shown.

| **Compound group** | **Compound class** | **Specifics** | **Associated standard** |
| --- | --- | --- | --- |
| hydrocarbons | alkane | C6 | Hexane |
|  |  | <C12 | 1-Octene |
|  |  | cyclic or ≥C12 | Camphene |
|  | alkene | ≤C5 | Isoprene |
|  |  | C6-C10 | 1-Octene |
|  |  | ≥C10 | Ocimene |
|  |  | cyclic | Camphene |
|  | benzenoids | all^*^ | Toluene |
| isoprene | isoprene | C5H8 | Isoprene |
| monoterpenes | monoterpenes | all^*^ | α-Pinene |
| oxygenated monoterpenes | oxygenated monoterpenes | all^*^ | Eucalyptol |
| sesquiterpenes | sesquiterpenes | all^*^ | β-Caryophyllene |
| oxygenated sesquiterpenes | oxygenated sesquiterpenes | all | Nerolidol |
| homoterpenes^1^ | homoterpenes | all | 1-Octene |
| green leaf volatiles | See specific list^2^ |  | See specific list^2^ |
| **oxygenated VOCs** | acid (=O, -OH) | <C9 | (Z)-3-Hexenyl acetate |
|  |  | ≥C9 | (Z)-3-Hexenyl butyrate |
|  | alcohol (-OH) | <C8 | (Z)-3-Hexen-1-ol |
|  |  | ≥C8 | 1-Octen-3-ol |
|  | aldehyde (=O, -H) | <C8, no C=C | Hexanal |
|  |  | <C8, with C=C | (E)-2-Hexen-1-al |
|  |  | C8 | Octanal |
|  |  | >C8 | Nonanal |
|  | ester (=O, -OR) | <C9 | (Z)-3-Hexenyl acetate |
|  |  | ≥C9 | (Z)-3-Hexenyl butyrate |
|  |  | cyclic/ ≥3 O | Methyl salicylate |
|  | furan | only 1 O | 2-Methylfuran |
|  |  | >1 O | 2-Furaldehyde |
|  | ketone (=O) | <C8 | 2-Butanone |
|  |  | C8 | Octanal |
|  |  | >C8 | Nonanal |
|  | oxygenated benzenoids | only 1 O | Benzaldehyde |
|  |  | >1 O | Methyl salicylate |
|  | other oxygenated VOCs^3^ | <C9 | (Z)-3-Hexenyl acetate |
|  |  | ≥C9 | (Z)-3-Hexenyl butyrate |
|  |  | cyclic/ ≥3 O | Methyl salicylate |
| **Other** | halogens (-Cl, -Br, -F) | all | Toluene |
|  | nitrogen-containing VOCs | all | Indole |
|  | other | all | Toluene |
|  | sulfur-containing VOCs | all | Toluene |

**^1^ Homoterpenes:**

(E)-3,8-dimethyl-1,4,7-nonatriene (**(E)-DMNT**) and (E,E)-4,8,12-trimethyltrideca-1,3,7,11-tetraene (**TMTT**).

**^2^ List of green leaf volatiles (GLVs)**

| ***Acids***  13(S)-hydroperoxy 9Z,11E,15Z-Octadecatrienoic acid (13-HPOTE)  13(S)-hydroperoxy 9Z,11E-octadecadienoic Acid (13-HPODE)  (E)-12-oxododec-10-enoic acid  9Z-traumatin  ***Alcohols***  n-hexanol  1-hexanol  (Z)-3-Hexen-1-ol  3-Hexen-1-ol  (Z)-3-Hexen-1-ol  (E)-2-Hexen-1-ol  ***Aldehydes***  acetaldehyde  4-Hydroperoxy-(E)-2-hexenal (HPHE)  4-Hydroxy-(E)-2-hexenal (HHE)  4-Oxo-(E)-2-hexenal (OHE)  n-Hexenal  2-Hexenal  (E)-2-Hexenal  3-Hexenal  (Z)-3-hexenal  ***Esters***  Hexyl acetate  n-Hexenyl acetate  (Z)-2-Hexenyl acetate | ***Esters continued…***  Z2-hexen-1-ol acetate  Z3-hexenyl acetate  Z-3-hexenyl acetate  3-Hexen-1-ol acetate  E-2-hexenyl acetate  Ethyl butyl acetate  Z3-Hexenyl propionate  n-Hexenyl butyrate  n-Hexyl butyrate  (E)-2-Hexenyl butyrate  (E)-3-Hexenyl butyrate  Z3-Hexenyl-a-methylbutyrate  Hexyl 2-methylbutyrate  (Z)-pent-2-enyl butyrate  n-Hexenyl valerate  E2-hexenyl isovalerate  Z3-hexenyl isovalerate  Acetic acid ethyl ester  Acetic acid hexyl ester  Hexadecanoic acid methyl ester  3-Methylbut-2-en-1-yl pivalate  Z3-hexenyl hexanoate  (E)-2-hexenyl hexanoate  Z3-hexen-1-ol formate  Methyl hexanoate  Ethyl hexanoate;  Methyl hexoate  Hexanoic acid, methyl ester  Hexanoic acid, ethyl ester |
| --- | --- |

**Compounds to (potentially) exclude from your analysis**

- Siloxanes (e.g., hexamethyl disiloxane, hexamethyl cyclotrisiloxane, octamethyl cyclotetrasiloxane)
- Phthalates (e.g., diethyl phthalate, dibutyl phthalate)
- Dibutyl adipate
- 2-Ethylhexyl salicylate
- Homosalate
- All compounds <C5 (e.g., acetone, ethyl acetate etc.), except C4 compounds containing oxygen (e.g., 2-butanone)
